# Association between weight-adjusted-waist index and α1-acid glycoprotein levels among adult females: Evidence from NHANES 2015–2020

**DOI:** 10.1097/MD.0000000000049088

**Published:** 2026-06-12

**Authors:** Qi Jia, Yaolong Liu, Liting Hao

**Affiliations:** a School of Medicine, Yan’an University, Yan’an, China; b Department of Traditional Chinese Medicine Neurology, Yan’an Traditional Chinese Medicine Hospital, Yan’an, China; c Department of Gastroenterology, Affiliated Hospital of Yan’an University, Yan’an, China.

**Keywords:** inflammation, NHANES, obesity, weight-adjusted-waist index, α1-acid glycoprotein

## Abstract

Obesity is a prevalent issue in daily life, and previous studies have demonstrated a correlation between inflammation and obesity. Additionally, some studies have investigated the relationship between inflammation and body mass index, a widely used indicator of obesity. In recent years, the weight-adjusted waist circumference index (WWI) has been introduced and increasingly utilized in the clinical assessment of obesity and overweight. However, its relationship with the inflammatory marker α1-acid glycoprotein (AGP) remains unclear. In this study, we analyzed data from 2899 adult women (mean age 33.42 ± 9.27 years) from the National Health and Nutrition Examination Survey (2015–2020). The WWI was calculated as the square root of waist circumference divided by body weight. Multiple linear regression analysis and smooth curve fitting were performed to validate the results. Finally, the relationship between WWI, AGP, and various clinical factors, including educational status, age, and hypertension incidence, was analyzed. Subgroup analysis and interaction tests were performed during the analysis. This study included 2899 adult female participants (mean age 33.42 ± 9.27 years). The mean levels of WWI and AGP were 10.97 ± 0.83 and 0.79 ± 0.24 g/L, respectively. In the fully adjusted model, multiple linear regression analysis revealed a significant positive correlation between WWI and AGP levels, with each unit increase in WWI associated with a 0.06 g/L increase in AGP (β = 0.06, 95% CI: 0.05–0.07). When WWI was categorized into quartiles, AGP levels in the highest quartile were 0.13 g/L higher than those in the lowest quartile (β = 0.13, 95% CI: 0.10–0.15). Moreover, smooth curve fitting identified an inflection point in this relationship at a WWI value of 11.78. In the adult female population in the United States, WWI and AGP exhibited a significant positive correlation.

## 1. Introduction

Obesity is a prevalent health issue and is clinically considered to be associated with excessive body fat. In recent years, obesity rates have been rising not only in the United States but also globally,^[1]^ making it a major public health concern. Beyond the physical limitations caused by obesity, clinical studies have established its association with various diseases, including well-known conditions such as diabetes, as well as certain types of cancer.^[2]^ Currently, more than 39.5% of adults in the United States are classified as obese or overweight.^[3]^ Moreover, obesity among the elderly remains a serious concern, with projections indicating that by 2030, over half of the elderly population will be obese or overweight.^[4]^ More concerningly, statistical analyses indicate that childhood obesity is an increasing issue. Furthermore, data indicate that childhood obesity rates have steadily risen in recent years, reaching approximately 18% in 2016.^[5]^ To accurately assess obesity, body mass index (BMI) is commonly used; however, this metric is influenced by various confounding factors and cannot distinguish between muscle and fat content.^[6,7]^ In 2018, the weight-adjusted waist index (WWI) was introduced as a novel indicator for assessing clinical obesity.^[8]^ It is considered more appropriate than the conventional BMI, and its calculation, based on waist circumference and body weight, is more accessible.

Alpha-1-acid glycoprotein (AGP) is a key acute-phase reactant whose circulating levels rise in response to systemic inflammation.^[9–14]^ Importantly, a Mendelian randomization study supports a causal relationship between elevated BMI and increased AGP levels, directly linking adiposity to this inflammatory marker.^[15]^ Prior research has further examined the association between AGP and general adiposity in female populations.^[16]^ However, BMI is a crude measure that does not distinguish fat distribution, particularly visceral adiposity, which is more closely tied to metabolic risk.

Numerous studies have shown that obese individuals may experience worsened metabolic dysfunction due to prolonged chronic inflammation.^[17,18]^ To facilitate early intervention for obesity-related complications, numerous studies have been conducted to investigate the relationship between obesity and inflammatory markers,^[19]^ including AGP, IL-6, and alpha-1 antichymotrypsin. However, BMI and waist circumference are the most commonly used metrics to assess their association with these inflammatory markers, yet BMI may introduce biases. Additionally, emerging metrics can more accurately identify individuals with abdominal obesity.^[20]^ However, no studies have specifically examined the correlation between WWI and AGP. Given this background, this study was designed to investigate the relationship between WWI and AGP.

## 2. Methods

### 2.1. Study population

**Figure 1.**
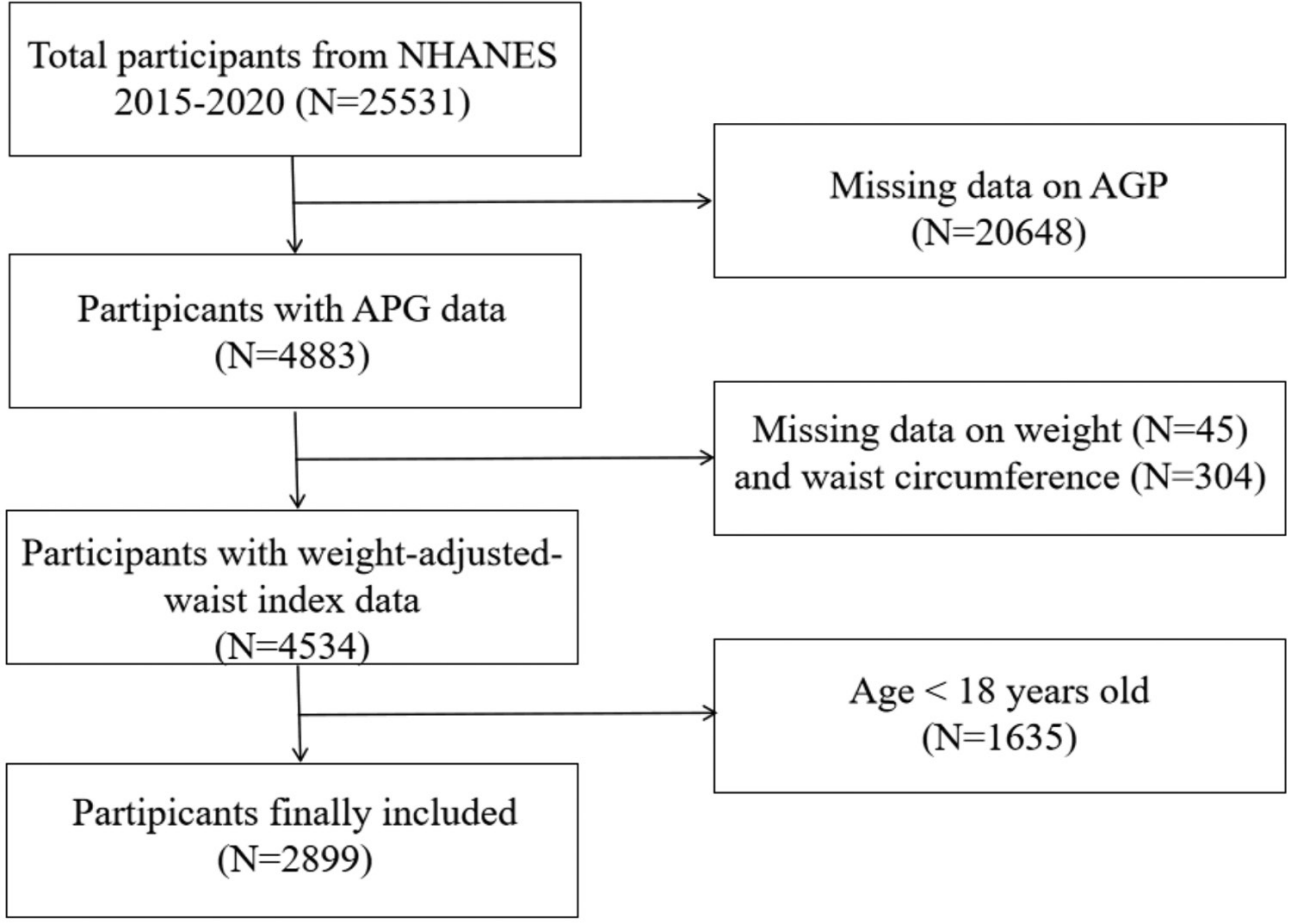
Flowchart of participant selection. AGP = α1-acid glycoprotein; NHANES = National Health and Nutrition Examination Survey; WWI = weight-adjusted-waist index.

Participants from the 2015 to 2020 National Health and Nutrition Examination Survey (NHANES) were selected. Of the 25,531 participants, 20,648 were excluded due to missing AGP data, 45 due to missing weight data, 304 due to missing waist circumference data, and 1635 due to being younger than 18 years of age, leaving 2899 adult females eligible for the study (Fig. 1).

### 2.2. Study variables

The relevant variables in this study included both independent and dependent variables, namely WWI and AGP. The covariates in this study included various indicators, including age, race/ethnicity, education level, the family poverty-income ratio (PIR), alanine transaminase (ALT), aspartate aminotransferase (AST), alkaline phosphatase (ALP), gamma-glutamyl transferase (GGT), total calcium, phosphorus, uric acid, total cholesterol, triglycerides, high-density lipoprotein cholesterol (HDL-C), and low-density lipoprotein cholesterol (LDL-C), hypertension, diabetes, smoking status (≥100 cigarettes in lifetime), moderate work activity, heavy alcohol consumption (≥4–5 drinks/day).

All analyses accounted for the complex, multi-stage probability sampling design of NHANES to generate nationally representative estimates. As our study variables incorporated data from household interviews, the Mobile Examination Center (MEC), and subsample laboratory measurements, we followed NHANES analytical guidelines. The appropriate subsample weights were applied. Specifically, the survey design was incorporated using the variables SDMVPSU (pseudo-primary sampling unit), SDMVSTRA (pseudo-stratum), and the 6-year MEC examination weight (WTMEC6YR) for the 2015–2020 cycles.

### 2.3. Statistical analysis

In this study, the data were statistically processed and analyzed, with statistical significance considered for *P* < .05. The variables were categorized into continuous and categorical variables. The variables were described using the mean ± standard deviation for continuous variables and percentages for categorical variables. Weighted multiple linear regression was applied to analyze the correlation between WWI and AGP, with the WWI levels of the observed subjects divided into four research groups. We constructed three progressively adjusted weighted linear regression models: Model 1 (Crude): unadjusted. Model 2 (Minimally adjusted): adjusted for basic demographic and socioeconomic factors: age, race/ethnicity, education level, and the family PIR. Model 3 (fully adjusted): additionally adjusted for an extensive set of potential confounders, including: Cardiometabolic conditions: hypertension, diabetes. Lifestyle factors: smoking status (≥100 cigarettes in lifetime), moderate work activity, heavy alcohol consumption (≥4–5 drinks/day). Serum biomarkers: ALT, AST, ALP, GGT, total calcium, phosphorus, uric acid, total cholesterol, triglycerides, HDL-C, and LDL-C. Covariates were selected a priori based on their established or plausible biological link to obesity, systemic inflammation, and/or AGP levels from the existing literature. The fully adjusted model (Model 3) aimed to isolate the independent association between WWI and AGP by accounting for a wide range of demographic, socioeconomic, behavioral, and clinical metabolic factors. We employed a complete-case analysis approach for the primary regression models. Participants with missing data on WWI, AGP, or any of the covariates specified in the fully adjusted Model 3 were excluded from that specific analysis. This ensures consistency and comparability across the nested models. The quartiles (Q1–Q4) of WWI were derived using weighted percentiles based on the NHANES examination weights (WTMEC6YR), ensuring the cut-points reflect the distribution in the target population (noninstitutionalized US adult females) rather than the simple sample distribution. The WWI and AGP levels of different groups were analyzed to examine the relationship between the two and observe their changing trends. Additionally, Subgroup analysis and interaction testing were performed to examine the potential correlation between the two, with subgroups including age, race, education, smoking at least 100 cigarettes, hypertension, and diabetes.

## 3. Results

### 3.1. Baseline characteristics

This study included 2899 female participants, with an average age of 33.42 ± 9.27 years. The mean WWI and AGP concentrations were 10.97 ± 0.83 and 0.79 ± 0.24 g/L, respectively. Characteristics of the participants were analyzed, as shown in Table 1. The study found that participants with higher AGP levels tended to be older and had a higher prevalence of hypertension and diabetes compared to those in other study groups. Levels of ALT, ALP, GGT, LDL cholesterol, total cholesterol, triglycerides, and uric acid were higher in the higher AGP group compared to the lower AGP group. In contrast, PIR, AST, blood phosphorus, and HDL cholesterol levels were lower. We also investigated AGP in the context of WWI quartile stratification and found that AGP levels significantly increased across WWI quartiles (Q1: 0.67 ± 0.20; Q2: 0.76 ± 0.21; Q3: 0.82 ± 0.23; Q4: 0.91 ± 0.24; *P*-trend < .001), as shown in Table 2.

**Table 1:** Baseline characteristics of participants in the NHANES from 2015 to 2020 (AGP quad-group n = 2899).

Characteristics	α1-acid glycoprotein				*P* value
	Q1 (N = 725)	Q2 (N = 723)	Q3 (N = 726)	Q4 (N = 725)	
Age (yr)	31.72 ± 9.30	33.24 ± 8.96	34.64 ± 9.35	34.07 ± 9.24	<.001
Race/ethnicity (%)					<.001
Non-Hispanic White	28.41	30.84	28.65	39.72	
Non-Hispanic Black	19.03	19.36	26.86	24.69	
Mexican American	14.07	19.64	19.01	14.62	
Other Hispanics	9.93	12.72	12.26	10.07	
Other races	28.55	17.43	13.22	10.90	
Education level (%)					<.001
<high school	11.72	15.21	17.91	14.62	
High school	12.69	18.12	19.83	20.83	
>High school	75.59	66.67	62.26	64.55	
Hypertension (%)					<.001
Yes	8.28	14.38	17.91	21.52	
No	91.72	85.62	82.09	78.48	
Diabetes (%)					
Yes	1.79	4.70	6.89	7.03	<.001
No	98.21	95.30	93.11	92.97	
Smoked at least 100 cigarettes (%)					
Yes	19.59	24.62	33.33	37.79	<.001
No	80.41	75.38	66.67	62.21	
Moderate work activity (%)					<.001
Yes	36.41	45.64	41.74	48.97	
No	63.59	54.36	58.26	51.03	
≥4–5 drinks/day (%)					<.001
Yes	3.45	5.53	6.89	8.69	
No	96.55	94.47	93.11	91.31	
PIR	2.63 ± 1.63	2.47 ± 1.55	2.18 ± 1.49	2.08 ± 1.47	<.001
ALT (U/L)	17.36 ± 21.58	17.92 ± 12.09	20.04 ± 16.38	19.91 ± 14.66	<.001
AST (U/L)	21.30 ± 32.40	19.88 ± 8.35	20.59 ± 12.92	20.27 ± 15.15	.04
ALP (IU/L)	56.95 ± 17.88	64.31 ± 17.66	71.28 ± 20.80	80.16 ± 24.19	<.001
GGT (IU/L)	16.59 ± 17.64	18.33 ± 15.22	24.33 ± 29.57	26.31 ± 36.16	<.001
Total calcium (mmol/L)	2.29 ± 0.08	2.30 ± 0.08	2.30 ± 0.09	2.30 ± 0.09	.48
Serum phosphorus (mmol/L)	3.71 ± 0.47	3.64 ± 0.51	3.62 ± 0.51	3.63 ± 0.55	.001
Uric acid (µmol/L)	242.05 ± 58.01	258.32 ± 58.57	274.32 ± 66.86	293.42 ± 75.29	<.001
Total cholesterol (mmol/L)	178.10 ± 36.10	179.17 ± 35.53	182.75 ± 37.80	180.28 ± 36.68	.03
HDL- cholesterol (mg/dL)	65.19 ± 15.33	58.12 ± 14.93	53.36 ± 14.92	49.19 ± 14.21	<.001
LDL- cholesterol (mg/dL)	101.15 ± 20.53	103.84 ± 21.45	105.22 ± 20.82	106.10 ± 19.61	<.001
Triglyceride (mg/dL)	81.29 ± 34.28	87.05 ± 44.23	92.55 ± 45.19	94.36 ± 39.68	<.001
WWI (cm/√kg)	10.53 ± 0.75	10.86 ± 0.76	11.11 ± 0.78	11.40 ± 0.79	<.001

Mean ± SD for continuous variables: the *P* value was calculated by the weighted linear regression model; (%) for categorical variables: the *P* value was calculated by the weighted chi-square test.

ALP = alkaline phosphatase, ALT = alanine transaminase, APG = α1-acid glycoprotein, AST = aspartate aminotransferase, GGT = gamma-glutamyl transferase, HDL-C = high-density lipoprotein, LDL-C = low-density lipoprotein, WWI = weight-adjusted waist index.

**Table 2:** Baseline characteristics of participants in the NHANES from 2015 to 2020 (WWI quad-group *n* = 2899).

Characteristics	WWI				*P* value
	Q1 (N = 725)	Q2 (N = 724)	Q3 (N = 724)	Q4 (N = 7260	
Age (yr)	29.45 ± 8.95	33.48 ± 9.43	34.93 ± 8.79	35.81 ± 8.62	<.001
Race/ethnicity (%)					<.001
Non-Hispanic White	36.69	32.46	27.49	30.99	
Non-Hispanic Black	25.38	20.99	21.27	22.31	
Mexican American	7.17	14.78	22.51	22.87	
Other Hispanics	10.76	11.60	12.02	10.61	
Other races	20.00	20.17	16.71	13.22	
Education level (%)					<.001
< high school	6.90	11.33	18.65	22.59	
High school	13.10	18.78	19.75	19.83	
> High school	80.00	69.89	61.60	57.58	
Hypertension (%)					<.001
Yes	6.48	11.88	17.13	26.58	
No	93.52	88.12	82.87	73.42	
Diabetes (%)					
Yes	0.28	2.62	6.22	11.29	<.001
No	99.72	97.38	93.78	88.71	
Smoked at least 100 cigarettes (%)					
Yes	22.34	27.76	31.77	33.47	<.001
No	77.66	72.24	68.23	66.53	
Moderate work activity (%)					.3
Yes	42.48	45.99	43.09	41.18	
No	57.52	54.01	56.91	58.82	
≥4–5 drinks/day (%)					.04
Yes	4.41	6.35	5.80	7.99	
No	95.59	93.65	94.20	92.01	
PIR	2.57 ± 1.64	2.47 ± 1.55	2.30 ± 1.50	2.03 ± 1.46	<.001
ALT (U/L)	15.38 ± 16.89	16.99 ± 9.88	20.18 ± 17.49	22.63 ± 19.49	<.001
AST (U/L)	19.22 ± 9.67	19.13 ± 6.44	21.18 ± 17.16	22.49 ± 32.84	.01
ALP (IU/L)	59.28 ± 16.92	64.93 ± 20.04	71.36 ± 23.06	77.06 ± 23.36	<.001
GGT (IU/L)	15.42 ± 11.69	17.98 ± 13.42	23.96 ± 36.44	28.19 ± 32.35	<.001
Total calcium (mmol/L)	2.31 ± 0.08	2.30 ± 0.08	2.29 ± 0.08	2.29 ± 0.09	.48
Serum phosphorus (mmol/L)	3.72 ± 0.48	3.66 ± 0.52	3.61 ± 0.52	3.62 ± 0.54	<.001
Uric acid (µmol/L)	248.33 ± 57.62	259.25 ± 61.57	270.11 ± 65.08	290.95 ± 77.79	<.001
Total cholesterol (mmol/L)	168.98 ± 31.10	180.14 ± 36.45	184.67 ± 38.03	186.52 ± 38.22	<.001
HDL-cholesterol (mg/dL)	62.18 ± 15.13	58.70 ± 15.81	54.59 ± 16.51	50.43 ± 13.96	<.001
LDL-cholesterol (mg/dL)	98.44 ± 19.09	104.05 ± 20.49	105.89 ± 21.40	107.93 ± 20.53	<.001
Triglyceride (mg/dL)	66.66 ± 23.01	75.65 ± 34.84	85.85 ± 47.13	90.80 ± 53.31	<.001
AGP	0.67 ± 0.20	0.76 ± 0.21	0.82 ± 0.23	0.91 ± 0.24	<.001

Mean ± SD for continuous variables: the *P* value was calculated by the weighted linear regression model; (%) for categorical variables: the *P* value was calculated by the weighted chi-square test.

ALP = alkaline phosphatase, ALT = alanine transaminase, APG = α1-acid glycoprotein, AST = aspartate aminotransferase, GGT = gamma-glutamyl transferase, HDL-C = high-density lipoprotein, LDL-C = low-density lipoprotein, WWI = weight-adjusted waist index.

### 3.2. Association between WWI and AGP

Table 3 presents the results of the multivariate linear regression analysis. In Model 1 (unadjusted model), WWI and AGP were significantly correlated (β = 0.11, 95% CI: 0.10–0.12). In Model 2, this relationship remained significant (β = 0.11, 95% CI: 0.10–0.12). Even in Model 3, the study found a significant positive correlation between WWI and AGP (β = 0.06, 95% CI: 0.05–0.07). Each unit increase in WWI was associated with a 0.06 unit increase in AGP score (β = 0.06, 95% CI: 0.05–0.07). Compared to the lowest quartile of WWI, AGP scores increased across higher WWI groups, with the highest quartile showing a 0.13 unit higher AGP score than the lowest quartile (β = 0.13, 95% CI: 0.10–0.15).

**Table 3:** Associations between weight-adjusted waist index and α1-acid glycoprotein.

WWI (cm/√kg)	AGP	
	β (95% CI)	*P* value
Crude model (Model 1)		
Continuous	0.11 (0.10, 0.12)	<.001
Categories		
Quartile 1	0 (ref)	
Quartile 2	0.09 (0.07, 0.12)	<.001
Quartile 3	0.16 (0.13, 0.18)	<.001
Quartile 4	0.24 (0.22, 0.27)	<.001
Minimally adjusted model (Model 2)		
Continuous	0.11 (0.10, 0.12)	<.001
Categories		
Quartile 1	0 (ref)	
Quartile 2	0.10 (0.07, 0.12)	<.001
Quartile 3	0.16 (0.14, 0.18)	<.001
Quartile 4	0.24 (0.22, 0.26)	<.001
Fully adjusted model (Model 3)		
Continuous	0.06 (0.05, 0.07)	<.001
Categories		
Quartile 1	0 (ref)	
Quartile 2	0.06 (0.04, 0.08)	<.001
Quartile 3	0.09 (0.07, 0.11)	<.001
Quartile 4	0.13 (0.10, 0.15)	<.001

Model 1: no covariates were adjusted. Model 2: age, race and education level were adjusted. Model 3: age, race, education level, PIR, ALT, AST, ALP, GGT, uric acid, total calcium, serum phosphorus, total cholesterol, HDL-cholesterol, LDL-cholesterol, triglyceride, hypertension, diabetes, smoked at least 100 cigarettes, moderate work activity, have 4/5 or more drinks every day were adjusted.

ALP = alkaline phosphatase, ALT = alanine transaminase, AST = aspartate aminotransferase, GGT = gamma-glutamyl transferase, HDL-C = high-density lipoprotein cholesterol, LDL-C = low-density lipoprotein cholesterol, PIR = ratio of family income to poverty, Q = quartile.

**Figure 2.**
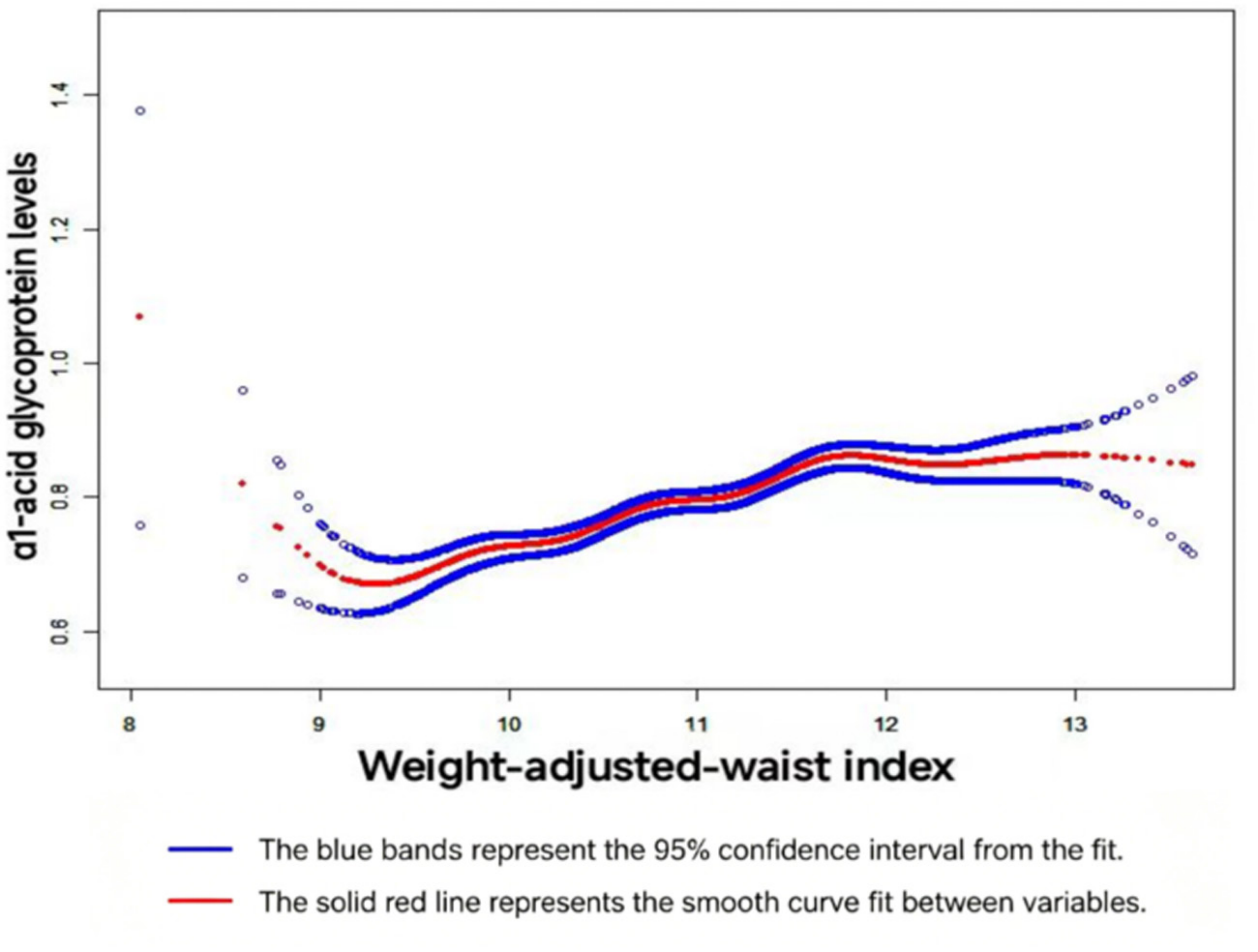
The association between WWI and AGP. (A) Each black point represents a sample. (B) The solid red line represents the smooth curve fit between variables. Blue bands represent the 95% confidence interval from the fit. AGP = α1-acid glycoprotein; WWI = weight-adjusted-waist index.

Additionally, further analyses using the smoothed curve fitting technique (Fig. 2) confirmed the association between WWI and AGP. After further calculation, the inflection point (*K*) was found to be 11.78. To the left of this point, a significant positive correlation was observed between WWI and AGP (β = 0.07, 95% CI: 0.06–0.08). However, no significant association was found between WWI and AGP on the other side (β = 0.00, 95% CI: −0.03–0.03) (Table 4).

**Table 4:** Threshold effect analysis of WWI on AGP using the two-piecewise linear regression model.

AGP (g/L)	Adjusted β (95% CI)
	*P* value
WWI	
Inflection point	11.78
WWI < 11.78	0.07 (0.06–0.08) < .0001
WWI > 11.78	0.00 (−0.03–0.03) .95
Log-likelihood ratio	<.001

Age, race, education level, PIR, ALT, AST, ALP, GGT, uric acid, total calcium, serum phosphorus, total cholesterol, HDL-cholesterol, LDL-cholesterol, triglyceride, hypertension, diabetes, smoked at least 100 cigarettes, moderate work activity, have 4/5 or more drinks every day were adjusted.

ALP = alkaline phosphatase, ALT = alanine transaminase, AST = aspartate aminotransferase, GGT = gamma-glutamyl transferase, HDL-C = high-density lipoprotein cholesterol, LDL-C = low-density lipoprotein cholesterol, PIR = ratio of family income to poverty, Q = quartile.

### 3.3. Subgroup analysis

This study analyzed the consistency of the relationship between AGP and WWI indicators in the population and examined potential differences. The participants in the study were further analyzed and categorized into several subgroups based on smoking (at least 100 cigarettes), diabetes, and hypertension (Table 5). The results indicated that the association between WWI and AGP was not significant in these subgroups (*P* > .05 for interaction).

**Table 5:** Subgroup analysis of the association between WWI and AGP.

Subgroup	AGP (β [95% CI])	*P* for interaction
Age (yr)		.40
18–19	0.04 (−0.00 to 0.07)	
20–29	0.06 (0.05 to 0.08)	
30–39	0.05 (0.03 to 0.07)	
40–49	0.06 (0.05 to 0.08)	
Diabetes		.54
Yes	0.07 (0.03 to 0.12)	
No	0.06 (0.05 to 0.07)	
Hypertension		.97
Yes	0.06 (0.04 to 0.08)	
No	0.06 (0.05 to 0.07)	
Race/ethnicity		.06
Non-Hispanic White	0.07 (0.05 to 0.08)	
Non-Hispanic Black	0.07 (0.05 to 0.09)	
Mexican American	0.04 (0.02 to 0.07)	
Other Hispanics	0.05 (0.02 to 0.07)	
Other races	0.03 (0.01 to 0.06)	
Education level		.05
< high school	0.03 (0.01 to 0.06)	
High school	0.05 (0.03 to 0.08)	
> high school	0.06 (0.05 to 0.08)	
Smoked at least 100 cigarettes		.24
Yes	0.05 (0.03 to 0.07)	
No	0.06 (0.05 to 0.07)	
Moderate work activity		.41
Yes	0.06 (0.05 to 0.08)	
No	0.05 (0.04 to 0.07)	

Age, race, education level, PIR, ALT, AST, ALP, GGT, uric acid, total calcium, serum phosphorus, total cholesterol, HDL-cholesterol, LDL-cholesterol, triglyceride, hypertension, diabetes, smoked at least 100 cigarettes, moderate work activity, have 4/5 or more drinks every day were adjusted.

ALP = alkaline phosphatase, ALT = alanine transaminase, AST = aspartate aminotransferase, GGT = gamma-glutamyl transferase, HDL-C = high-density lipoprotein cholesterol, LDL-C = low-density lipoprotein cholesterol, PIR = ratio of family income to poverty, Q = quartile.

## 4. Discussion

In this study, we conducted a cross-sectional analysis of 2899 participants and observed an association between WWI and AGP. Specifically, higher WWI values were associated with higher levels of AGP. Notably, an inflection point (*K*) of 11.78 was found between WWI and AGP, below which a positive association was observed. In conclusion, the WWI index can be considered a valuable indicator for assessing AGP levels.

Previous studies have investigated the relationship between inflammatory factors and obesity. This study introduces an innovative approach by analyzing the relationship between WWI and AGP while selecting different indicators and target populations to assess obesity and inflammatory responses. Yu et al conducted a correlation analysis using Systemic Immune-Inflammation Index (SII) and BMI as research indicators and confirmed a significant positive association.^[21]^ Additionally, researchers have identified a nonlinear inverted *U*-shaped relationship between SII and BMI, where a negative correlation appears beyond the inflection point. This phenomenon may be related to obesity-induced complications or inflammation-driven metabolic disorders leading to weight loss. Similarly, Zhou et al found a significant association between higher levels of SII/Systemic inflammatory response index and obesity, with a stronger correlation observed.^[22]^ Inflammatory factors and obesity are closely intertwined. As researchers continue to explore obesity-related diseases and inflammatory cell responses, the concept of “meta-inflammation” was introduced to describe the chronic inflammatory state associated with obesity.^[23]^ As early as 1993, Hotamisligil et al reported elevated tumor necrosis factor-alpha levels in obese individuals.^[24]^ More recently, Ferrari et al conducted a study on healthy European children and found a significant relationship between BMI and inflammatory markers such as AGP.^[25]^ Similarly, Sobieska et al observed elevated AGP levels in obese participants in a cohort study of Polish children aged 12–18 years.^[26]^ The findings of the present study align with previous research, further suggesting that obese or overweight individuals may experience persistent systemic inflammation. The relationship between WWI, as a novel body mass assessment index, and AGP holds significant clinical importance. Since WWI requires only body weight and waist circumference measurements, it serves as a more practical and accessible alternative to traditional indicators such as BMI for evaluating obesity-related inflammation. This enables early detection and facilitates the development of preventive strategies to mitigate the risk of multi-organ complications resulting from chronic inflammatory conditions in obese individuals.

The relationship between WWI and AGP remains insufficiently explored, despite numerous animal studies identifying mechanisms linking obesity or overweight to inflammation.^[27,28]^ On one hand, prior research suggests a link between the dietary habits of obese individuals and increased intestinal permeability, leading to elevated levels of gram-positive bacterial lipopolysaccharide (LPS). This, in turn, activates pattern recognition receptors, triggering an inflammatory response.^[29]^ Some researchers argue that LPS primarily acts as an amplifier of systemic inflammation rather than a direct trigger of tissue inflammation. Additionally, free fatty acids in obese patients may indirectly promote inflammation via TLR4 and TLR2, leading to the activation of NF-κB and JNK1 and ultimately resulting in the infiltration of pro-inflammatory macrophages.^[30]^ To verify this, experiments on TLR4-deficient mice demonstrated an absence of obesity-induced inflammatory responses.^[30]^ Similarly, TLR2 knockout models exhibited protection against insulin resistance.^[31,32]^ On the other hand, adipose tissue macrophages (ATMs) play a crucial role. Upon LPS stimulation, macrophages transition to the M1 (pro-inflammatory) state, initiating the subsequent obesity-related Th1 response. Conversely, Th2-associated factors promote macrophage activation into the M2 state, which further modulates NF-κB-related responses. Interestingly, ATMs exhibit dynamic M1/M2 states depending on nutritional status. However, obesity or overweight conditions drive ATMs to shift from the M2 state to the pro-inflammatory M1 state.^[33–35]^ While the aforementioned cytokines and pathways are pro-inflammatory, adipose tissue also secretes antiinflammatory factors such as lipocalin, alongside pro-inflammatory mediators in obese individuals.^[36,37]^ Obese or overweight individuals exhibit lower lipocalin levels than their healthy or lean counterparts.^[38]^ As an antiinflammatory factor, lipocalin’s activity is modulated by pro-inflammatory signals.^[39]^ Clinical observations further indicate a negative correlation between lipocalin levels and visceral fat accumulation.^[38]^

This study has several limitations. First, the cross-sectional design precludes the establishment of causal inferences between WWI and AGP. Second, reliance on AGP as a single inflammatory marker limits the generalizability of our findings to broader inflammatory states. Although C-reactive protein and lipocalin have been strongly linked to obesity,^[40,41]^ incorporating a panel of markers in future studies would provide a more comprehensive assessment. Third, despite extensive adjustment for covariates, residual confounding by unmeasured or imprecisely measured factors cannot be excluded. Fourth, the definition of obesity in this analysis is based on anthropometric indices and was not audited against clinical diagnoses or body composition measurements, which may introduce measurement error. Fifth, the study sample was restricted to U.S. adult females, limiting the exploration of these associations in males and other populations. Finally, missing AGP data led to the exclusion of some participants, although we mitigated this by using multiple survey cycles to ensure a robust sample size. Despite these limitations, this study provides novel insights into the association between a refined adiposity index and systemic inflammation in a large, nationally representative cohort. However, this was mitigated by extending the study period, ensuring a large and representative sample of women. Interaction analysis can be employed to validate the potential association between WWI and AGP.

### 4.1. Public health implications

WWI measurement is accessible and straightforward from a public health perspective, requiring only a tape measure and a scale. This simplicity makes it a valuable tool for large-scale screening and community health initiatives. The strong association between WWI and AGP, an inflammatory marker, indicates that WWI may be integrated into routine health assessments or public health surveys. As a low-cost indicator, WWI can help identify individuals at risk of obesity-related chronic inflammation, enabling early, population-level risk stratification and informing targeted health campaigns.

## 5. Conclusion

This study suggests a significant positive association between the WWI and AGP levels among adult females in the United States. The finding of a nonlinear relationship also points to a possible threshold effect. These results add to evidence linking central adiposity, measured by the new WWI metric, with systemic inflammation. In clinical and public health practice, direct assessment of body composition is often impractical. WWI may instead serve as a simple and cost-effective way to identify those at risk of obesity-related chronic inflammation. Future longitudinal studies should confirm the causal direction of this relationship and test its usefulness in different populations and healthcare settings.

## Acknowledgments

We would like to thank all participants in this study.

## Author contributions

**Conceptualization:** Qi Jia, Liting Hao.

**Data curation:** Qi Jia, Liting Hao.

**Formal analysis:** Qi Jia, Liting Hao, Yaolong Liu.

**Funding acquisition:** Qi Jia, Liting Hao, Yaolong Liu.

**Investigation:** Qi Jia, Liting Hao, Yaolong Liu.

**Methodology:** Qi Jia, Liting Hao.

**Project administration:** Qi Jia.

**Resources:** Qi Jia.

**Software:** Qi Jia.

**Supervision:** Qi Jia.

**Validation:** Qi Jia.

**Visualization:** Qi Jia.

**Writing – original draft:** Qi Jia.

**Writing – review & editing:** Qi Jia.
